# Pediatric renal pseudotumour

**DOI:** 10.1259/bjrcr.20200088

**Published:** 2020-11-19

**Authors:** David Ola, Ravikumar Hanumaiah, Anand Majmudar

**Affiliations:** 1 Department of Radiology, SUNY Upstate Medical University Syracuse, New York, NY, USA

## Abstract

We present a case of 6-year-old female with history of respiratory distress who went into respiratory failure requiring intubation. Patient was subsequently found to be in hypertensive crisis with hyponatremic hypochloremic metabolic acidosis and acute kidney injury. Renal ultrasound was performed to find the cause of hypertension. The ultrasound study demonstrated lobulated isoechoic to hyper echoic mass-like lesion in the middle and lower pole of the right kidney with increased vascularity on Color Doppler examination. The renal mass was finally diagnosed as a pseudotumour, representing hypertrophied portion of the spared normal renal parenchyma in otherwise atrophic right kidney. Diagnosis was made using a combination of US, MRI, DMSA and CT angiography thus avoiding unnecessary surgical intervention.

## Clinical history

A 6-year-old female was admitted from an outside hospital with hypertensive crisis, right-sided pneumonia, status post respiratory failure and cardiac arrest with return of spontaneous circulation. Laboratory parameters demonstrated hyponatremic hypochloremic metabolic acidosis and acute kidney injury consistent with mixed nephritic and nephrotic syndrome.

## Imaging findings

**Figure 1.**
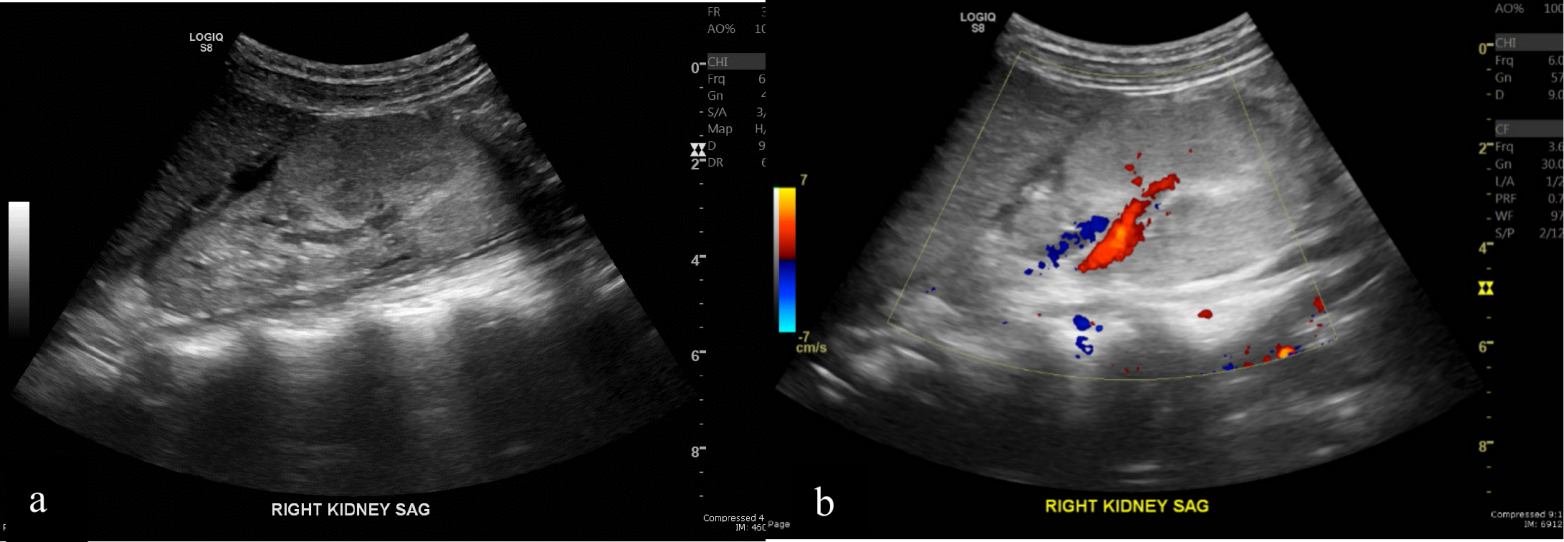
(a). Longitudinal ultrasound (US) of the right kidney demonstrating a lobulated isoechoic to hyperechoic mass-like area in the middle and lower pole (b). Longitudinal colour Doppler US image of right kidney demonstrates blood supply within the mass.

On admission, ultrasound (US) and Renal Doppler evaluation were performed using GE S8 portable us scanner with 2–9 MHz. Renal us revealed a 3.4×4.8×5.2 cm lobulated isoechoic to hyperechoic mass-like lesion with increased vascularity in the middle and lower pole of the right kidney (Figure 1). Based on the us findings, a renal tumour such as Wilms tumour or Angiomyolipoma was considered as differential.

**Figure 2.**
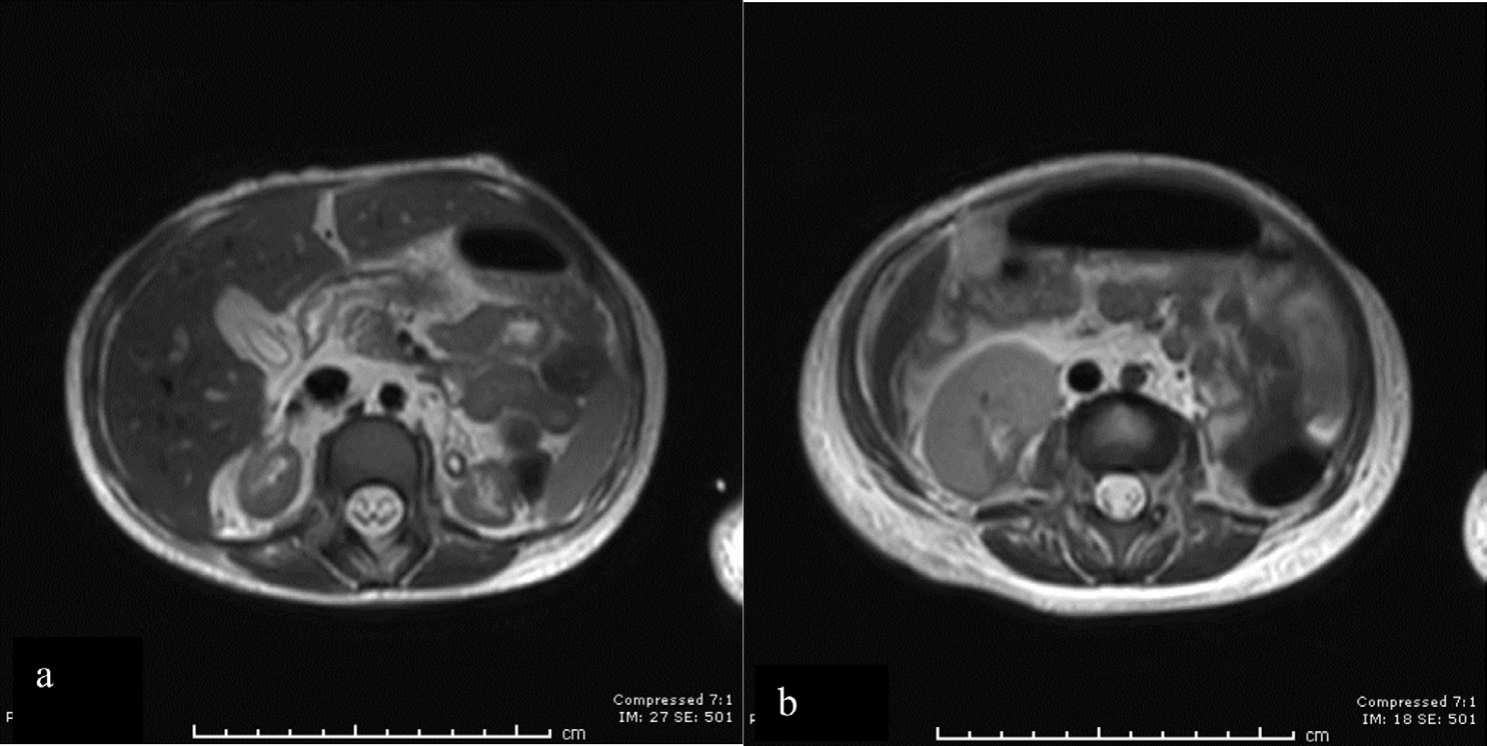
(a). Axial view *T*_2_-weighted MRI demonstrating atrophic upper pole of both the kidneys. (b). Axial view *T*_2_-weighted MRI demonstrating lobulated T2 hyperintense signal in the middle and lower pole of the right kidney following renal contour.

**Figure 3.**
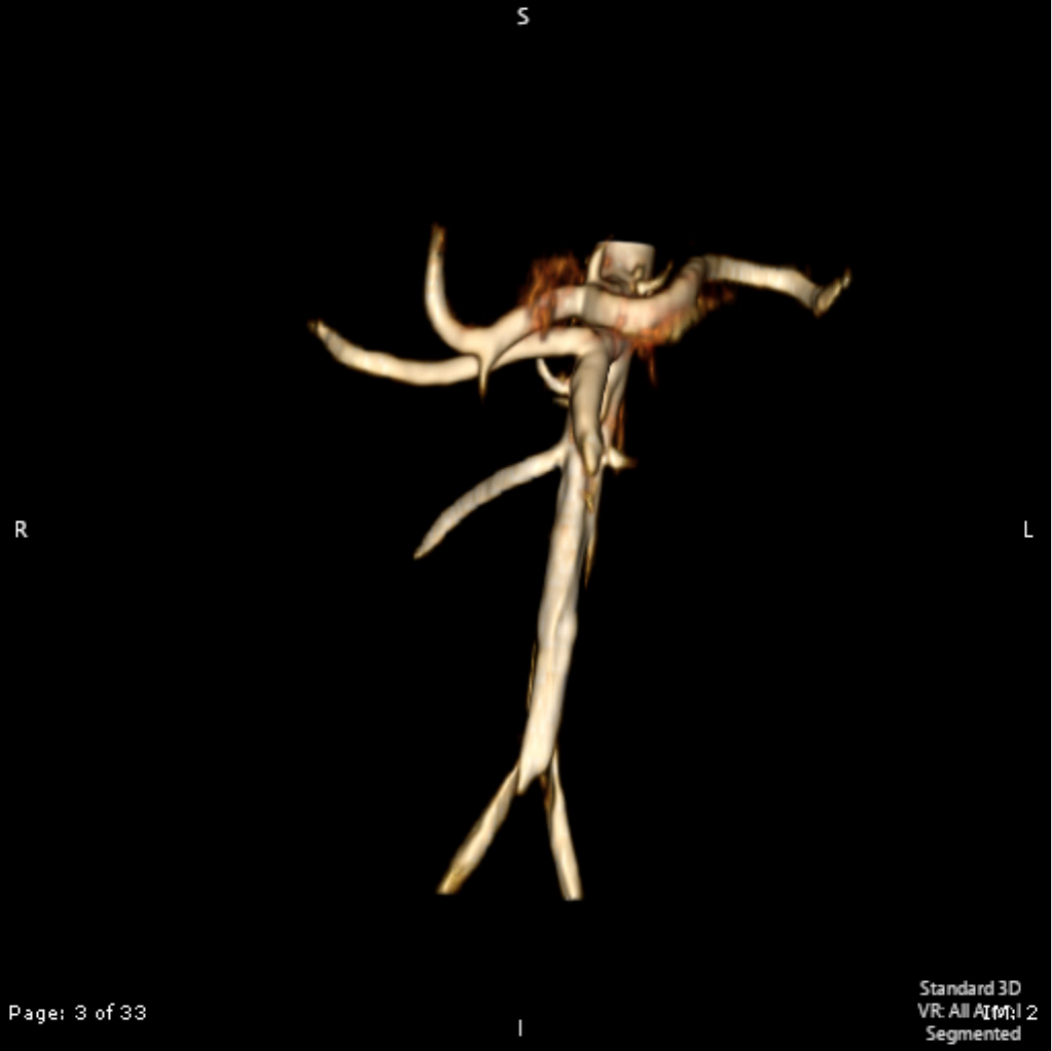
3D reconstructed image demonstrating right renal artery supplying the lower pole of the right kidney. Non-contrast MRA volume-rendered image demonstrating prominent right renal artery supplying the pseudotumour/lower pole of the right kidney.

Two days following presentation, MRI and magnetic resonance angiography (MRA) were performed without contrast on a 3 T unit (Philips). MRI demonstrated bilaterally atrophic kidneys and confirmed the presence of a mass like lesion at the right lower pole. The mass demonstrated slightly T2 hyperintense and T1 isointense signal with no significant restricted diffusion (Figure 2). A branch of the right renal artery supplied the renal mass (Figure 3). The rest of the right renal parenchyma was atrophic. The right renal artery was well delineated with no evidence of renal artery stenosis. The left renal artery was diminutive, however with no definite evidence of focal narrowing or stenosis. At this point, renal artery stenosis was considered less likely but renal neoplasm remained under consideration. Plasma metanephrines and urine homovanillic acid/vanillylmandelic acid were unremarkable. The clinical decision was made to consider the need for nephrectomy, using Wilms’ tumour suppressor gene1 (WT1 gene) testing to help guide surgical decisions.

**Figure 4.**
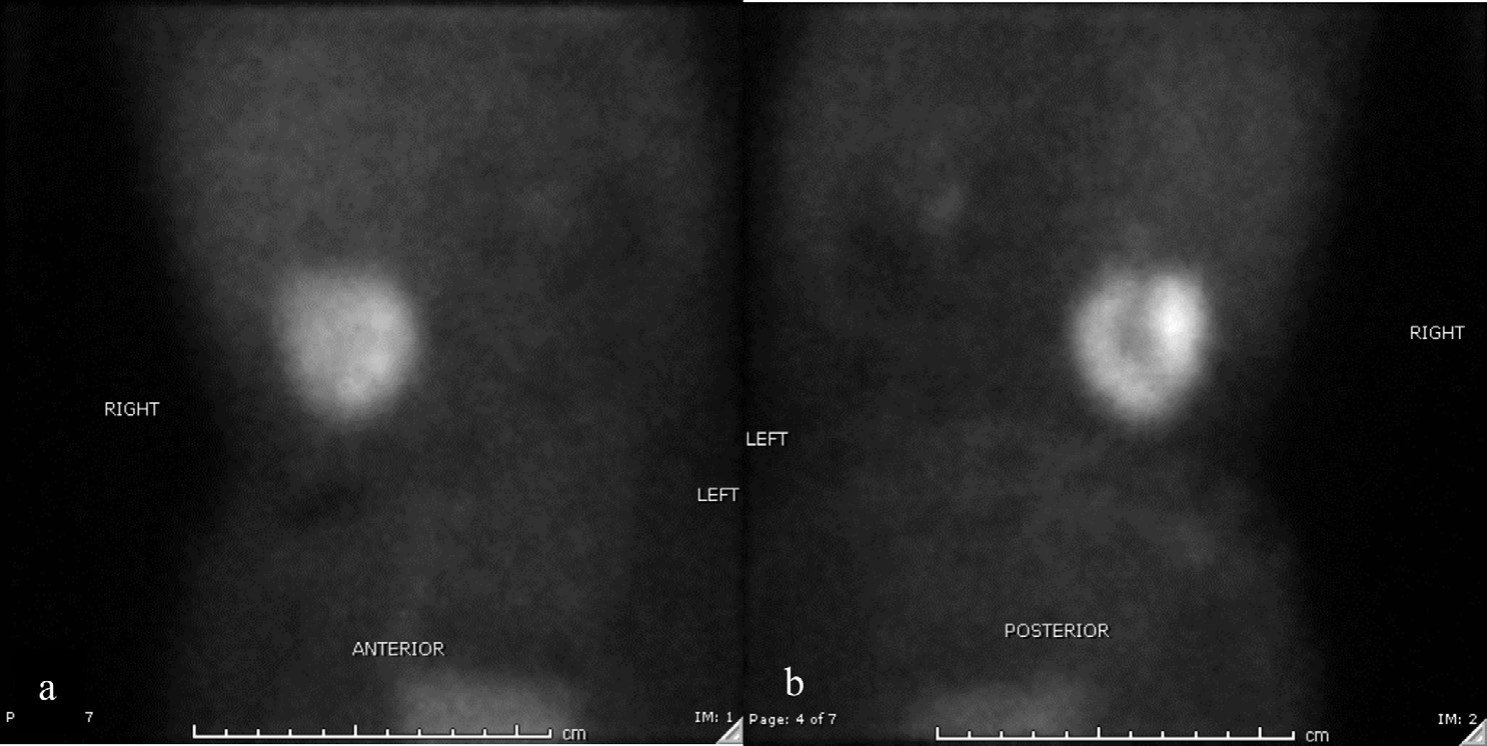
^99m^Tc-DMSA scintigraphy demonstrating increased activity in the lower pole of right kidney corresponding to the lobulated mass-like area of the lower pole of the right kidney. (a) Anterior view, (b) Posterior view.

25 days after presentation, dimercaputo succinic acid (DMSA) scan was performed to evaluate for functional anatomy of kidneys before possible surgery (partial nephrectomy). Posterior projection images of the kidneys were acquired 2 h after injection of 0.913 millicurie (mCi) of Tc-99m labelled DMSA. ^99m^Tc-DMSA scintigraphy showed no activity in the left kidney and the upper pole of the right kidney. However, it showed increased activity at the lower pole of the right kidney with demonstrable cortical outline (Figure 4). At this point, possibility that the presumed renal mass was normal functional renal tissue and the remainder of the right kidney and entire left kidney being dysplastic was considered. The WT1 testing was negative which made possibility of mass being Wilms’ tumour less likely. Biopsy was not performed as the previous investigations were less favourable for a mass and due to risk of bleeding and potential damage to functional renal tissue.

**Figure 5.**
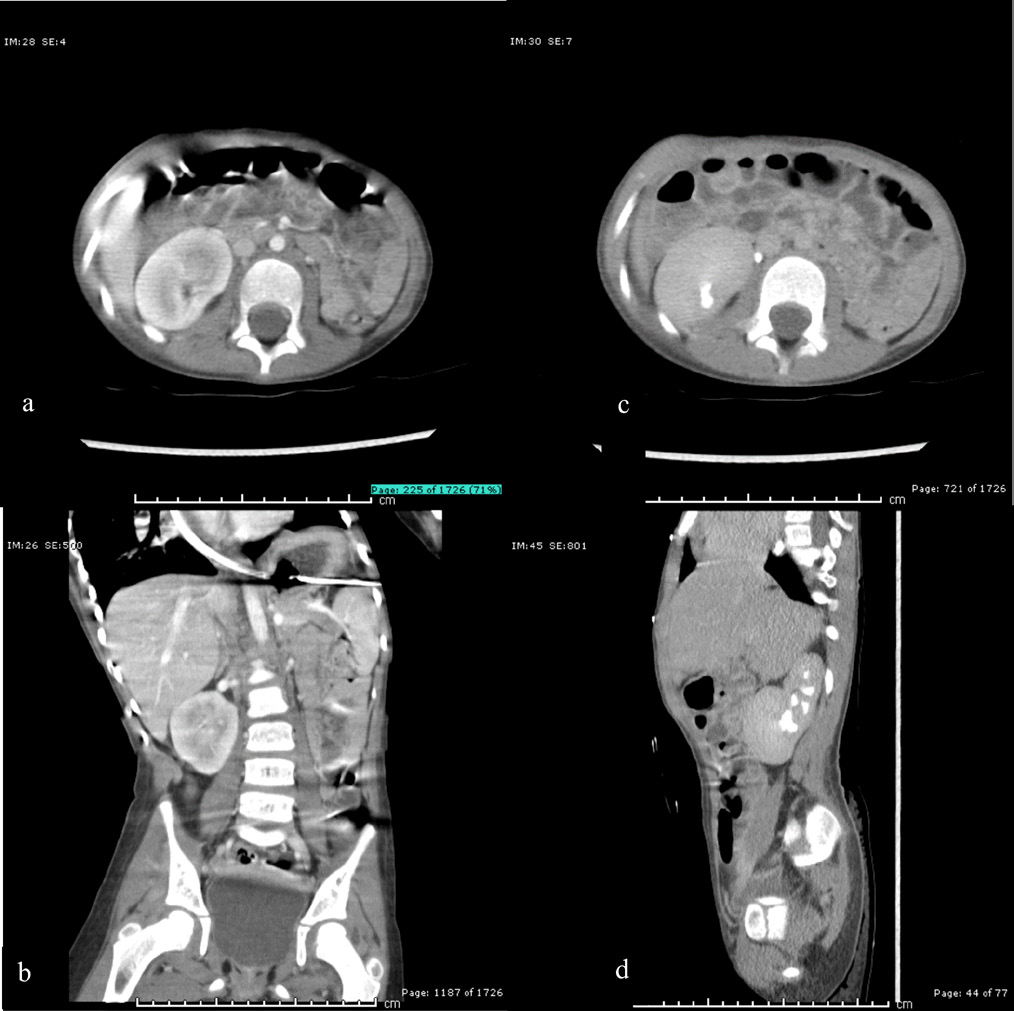
(a) Axial view, (b) Coronal view. CT image of abdomen demonstrating normal cortical enhancement of the right lower pole of the right kidney. (c) Axial view, (d) Sagittal view. CT image of abdomen demonstrating excretion of contrast into the lower calyx of the right kidney.

A week after DMSA scan, CT angiography (CTA) was performed in order to evaluate the perfusion of right kidney and excretion of contrast from right kidney. CTA scan of abdomen was done with i.v. contrast on Light speed VCT 64 slice scanner. CTA demonstrated marked atrophic appearance of the left kidney and the upper pole of the right kidney. CTA also confirmed a persistent solid mass-like lesion arising from the lower pole of the right kidney with homogeneous enhancement on the venous phase images and preservation of normal corticomedullary differentiation (Figure 5). Given the imaging appearance and apparent preserved functionality of the lower pole of right kidney, the mass was determined to be a pseudotumour most likely due to hypertrophy of the spared normal renal parenchyma. Prior to discharge, blood pressure normalized in the intensive care unit with the use of i.v. anti-hypertensives and the renal function also improved with creatinine declining to 1.2 from 2.2 mg dl^−1^. On follow-up, blood pressure and renal function continued to remain stable.

## Discussion

Renal pseudotumours should be considered in the differential diagnosis of renal masses. Renal pseudotumours can be congenital (*e.g.* prominent columns of Bertin and dromedary hump), infectious (focal pyelonephritis and renal abscess), vascular (arteriovenous malformation) or acquired (hypertrophic renal parenchyma).^1^

Renal pseudotumour due to hypertrophic compensatory changes have been reported in an adult patient with bilateral renal artery stenosis, but also with a patent accessory renal artery to the spared parenchyma in a patient with bilateral atrophic kidneys.^2^ The diagnosis was made with radiological imaging but also required ultrasound-guided biopsy with histological studies.

We report a case of a renal mass in a paediatric patient with bilateral atrophic kidneys with no evidence of renal artery stenosis. The renal mass was a pseudotumour secondary to compensatory hypertrophy of spared normal renal parenchyma in a patient with otherwise atrophic kidneys. Reported cases of renal pseudotumours due to compensatory hypertrophy of the kidneys are rare and typically require biopsy for diagnosis.^2^ We were able to diagnose renal pseudotumour due to compensatory hypertrophy in a paediatric patient using a combination of renal US, MRI, ^99m^Tc-DMSA scintigraphy and CT without biopsy.

Renal pseudotumours can also be due to other etiologies such as fetal lobulation, renal hilar lip, cross-fused ectopia and IgG4-related disease (IgG4-RD). Persistent fetal lobulation typically present with smooth indentations of the renal outline that overlie the space between the pyramids.^1^ Renal hilar lip presents with an in folding of the cortex at the level of the renal sinus.^3^ Persistent fetal lobulation and renal hilar lip are variation of normal renal parenchyma with normal renal function and do not present with an accessory renal artery. Crossed fused ectopia presents with fused kidneys located on the same side.

IgG four related disease (IgG4-RD) is a systemic fibroinflammatory disease affecting different tissues or organs characterized by tumour-like infiltration of IgG4-positive plasma cells, often with elevated plasma levels of IgG4. Renal manifestations of IgG4-RD include idiopathic hypocomplementic tubulointerstitial nephritis, multifocal fibrosclerosis and inflammatory pseudotumour. The disease is very rare in paediatric population. A systematic review found 25 cases in children with a median age of 13 years and female predominance, in contrast to the male predominance in the adult disease.^4^ In the paediatric population, ophthalmic IgG4-RD was the most common entity followed by autoimmune pancreatitis.^4^ Although we did not obtain plasma levels of IgG4, IgG4-RD is less likely given the absence of multi-organ involvement. Finally, none of these etiologies are typically associated with bilateral atrophic kidneys.

Our literature search revealed one other case of paediatric compensatory renal hypertrophy presenting as a pseudotumour, diagnosed with multimodal radiological imaging without biopsy.^5^ Compensatory renal pseudotumours should be considered in patients presenting with renal mass in otherwise atrophic kidneys.

## Learning points

1. Renal pseudotumours may mimic renal neoplasms on imaging and should be considered in differential.
2. Diagnosis of renal pseudotumour can be done with combination of US, MRI, Tc99 DMSA without or with CT angiography thus eliminating the need of biopsy.
3. Clinically, patients with renal pseudotumour due to compensatory hypertrophy may present with impaired renal function since the kidneys are otherwise atrophic.
4. Presence of corticomedullary differentiation in the pseudotumour helps in identifying normal renal parenchyma, and ruling out a true neoplasm.
